# Performance of penalized maximum likelihood in estimation of genetic covariances matrices

**DOI:** 10.1186/1297-9686-43-39

**Published:** 2011-11-27

**Authors:** Karin Meyer

**Affiliations:** 1 Animal Genetics and Breeding Unit, University of New England, Armidale NSW 2351, Australia

## Abstract

**Background:**

Estimation of genetic covariance matrices for multivariate problems comprising more than a few traits is inherently problematic, since sampling variation increases dramatically with the number of traits. This paper investigates the efficacy of regularized estimation of covariance components in a maximum likelihood framework, imposing a penalty on the likelihood designed to reduce sampling variation. In particular, penalties that "borrow strength" from the phenotypic covariance matrix are considered.

**Methods:**

An extensive simulation study was carried out to investigate the reduction in average 'loss', i.e. the deviation in estimated matrices from the population values, and the accompanying bias for a range of parameter values and sample sizes. A number of penalties are examined, penalizing either the canonical eigenvalues or the genetic covariance or correlation matrices. In addition, several strategies to determine the amount of penalization to be applied, i.e. to estimate the appropriate tuning factor, are explored.

**Results:**

It is shown that substantial reductions in loss for estimates of genetic covariance can be achieved for small to moderate sample sizes. While no penalty performed best overall, penalizing the variance among the estimated canonical eigenvalues on the logarithmic scale or shrinking the genetic towards the phenotypic correlation matrix appeared most advantageous. Estimating the tuning factor using cross-validation resulted in a loss reduction 10 to 15% less than that obtained if population values were known. Applying a mild penalty, chosen so that the deviation in likelihood from the maximum was non-significant, performed as well if not better than cross-validation and can be recommended as a pragmatic strategy.

**Conclusions:**

Penalized maximum likelihood estimation provides the means to 'make the most' of limited and precious data and facilitates more stable estimation for multi-dimensional analyses. It should become part of our everyday toolkit for multivariate estimation in quantitative genetics.

## Introduction

Estimation of genetic parameters, i.e. the partitioning of phenotypic variation into (co)variances due to genetic effects and other sources, is one of the basic tasks in quantitative genetics. Increasingly, livestock improvement schemes consider a multitude of traits. In turn, this requires complex, multivariate analyses that consider more than just a few traits simultaneously. Advances in modelling, improvements in computational algorithms and of corresponding software, paired with the capabilities of modern computer hardware have brought us to a point where large-scale analyses comprising numerous traits and records on tens of thousands of individuals are feasible. For example, Tyrisevä et al. [1] recently presented multivariate analyses for 25 traits, more than 100 000 sires and up to 325 parameters to be estimated. However, comparatively little attention has been paid to the problems associated with sampling variation that are inherent in multivariate analyses, which increase dramatically with the number of traits and the number of parameters to be estimated.

It has long been known that the eigenvalues of estimated covariance matrices are over-dispersed, i.e. that the largest sample eigenvalues are systematically biased upwards and the smallest values are biased downwards, while their mean is expected to be unbiased [2]. Moreover, a large proportion of the sampling variances of estimates of individual covariances can be attributed to this excess dispersion [3]. This is the more pronounced the larger the matrix, the smaller the data set and the more similar the population eigenvalues are. Hill and Thompson [4] demonstrated how this affected estimates of genetic covariance matrices and that it resulted in high probabilities of obtaining non-positive definite estimates. While maximum likelihood (ML) based methods of estimation make efficient use of all the data and readily allow estimates of covariance matrices to be constrained to the parameter space [5], the problems of sampling variation remain. Even multivariate analyses based on relatively large data sets are thus likely to yield imprecise estimates. Furthermore, we have scenarios where the numbers of records are invariably limited. This includes data for new traits or traits which are 'hard to measure', e.g. carcass characteristics of meat producing animals. Similarly, evolutionary biologists concerned with quantitative genetics of natural populations are usually restricted to rather small samples.

Hence, any avenue to 'improve' estimates, i.e. to obtain estimates which are on average closer to the population values, should be given serious consideration. To begin with, we have accumulated a substantial body of knowledge about genetic parameters for various traits. However, typically this is not used. While the Bayesian paradigm directly provides the means to incorporate such prior information, applications in estimating covariance components often assume flat or uninformative priors [6], i.e. do not fully exploit its advantages. Secondly, multivariate covariance matrices can often be modelled parsimoniously by imposing some structure. This decreases sampling variation by reducing the number of parameters to be estimated. Common examples are factor-analytic and reduced rank models or treating covariance matrices as 'separable', i.e. as the direct product of two or more smaller matrices (see Meyer [7] for a detailed review). Finally, statistical techniques are available - often referred to as regularization methods - which substantially reduce sampling variance, albeit at the expense of introducing some bias, and thus yield 'better' estimates. Interest in regularized estimation for multivariate analyses and the trade-off between sampling variance and bias dates back to the 1970's and earlier, stimulated in particular by the work of Stein, e.g. [8,9]. Recently, applications involving estimation in very high-dimensional settings have attracted resurgent attention, in particular for genomic data, e.g. [10-13].

However, there has been little interest in regularized estimation in estimating genetic parameters. An early proposal, due to Hayes and Hill [14], was to shrink the canonical eigenvalues in a one-way analysis of variance towards their mean and thus to reduce sampling variation. This yielded an estimate of the genetic covariance matrix that was a weighted combination of the standard (i.e. not regularized) estimate and the phenotypic covariance matrix multiplied by the mean eigenvalue. The authors thus described their method as 'bending' the genetic towards the phenotypic covariance matrix. A simulation study demonstrated that 'bending' could substantially increase the achieved response to selection based on an index derived using the modified estimates [14]. However, 'bending' has found little application except to force covariance matrices obtained by pooling estimates from multiple sources to be positive definite.

Recently, Meyer and Kirkpatrick [15] proposed to employ penalized restricted maximum likelihood (REML) to estimate genetic covariance matrices, and showed that imposing a penalty proportional to the variance among the canonical eigenvalues acted analogously to 'bending'. They demonstrated by simulation that this greatly reduced sampling and mean square errors, and, moreover, that this held for animal model analyses with a complicated pedigree structure and many different types of covariances between relatives. This paper extends the approach of Meyer and Kirkpatrick [15] to different types of penalties and, based on an extensive simulation study, examines various strategies to determine the amount of penalization to be applied.

## Penalized maximum likelihood estimation

### Improved estimation

The quality of a statistical estimator is generally quantified by some measure of the difference between the estimator and the true value, or *loss*. A well known quantity is the mean square error which is a quadratic loss, comprised of the sampling variance and the square of the bias in the estimator. We talk about improving an estimator when we are able to modify it in some way so that it has reduced loss, i.e. is closer to the true value. Usually this involves a trade-off between a reduction in sampling variance and additional bias. For covariance matrices, commonly employed measures of divergence are the entropy (*L*_1_) and quadratic (*L*_2_) loss [8]:

(1) $$\begin{array}{c} L_{1}\left(\Sigma ,\hat{\Sigma }\right)=\text{tr}\left(\Sigma^{-1}\hat{\Sigma }\right)-\log|\Sigma^{-1}\hat{\Sigma }|-q\;\text{and}\\ L_{2}\left(\Sigma ,\hat{\Sigma }\right)=\text{tr}{\left(\Sigma^{-1}\hat{\Sigma }-I\right)}^{2} \end{array}$$

where **Σ **and $\hat{\Sigma }$ denote a covariance matrix of size *q *× *q *and its estimator, respectively, and *q *represents the number of traits.

A reduction in loss can often be achieved by regularizing estimators. In broad terms, *regularization *describes a scenario where estimation for somewhat ill-posed or overparameterized problems is improved through use of some form of additional information. Frequently the latter involves a penalty for the deviation from a desired outcome. For example, in modelling curves using splines a 'roughness penalty' is employed to place preference on simple, smooth functions [16]. Well known forms of regularization are ridge regression [17] and the LASSO (Least Absolute Shrinkage and Selection Operator) [18]. Whilst these methods were originally developed to encourage shrinkage of regression coefficients, corresponding applications for the estimation of high-dimensional covariance matrices have been described; see Meyer and Kirkpatrick [15] for a review and references.

### Penalizing the likelihood

In Bayesian estimation, some degree of regularization is 'built in' through the specification of a prior and the associated degree of uncertainty. In a ML framework, either 'full' ML or REML, prior information can be incorporated by penalizing the likelihood. A general way to select a penalty is to specify a prior distribution for the parameters to be estimated for a suitable choice of parameterisation. The penalty is then obtained as minus the logarithmic value of the density of the prior, and a so-called tuning factor determines the relative emphasis to be given to the data and the penalty. In the following, we consider penalized REML estimation for two categories of penalties: those which are a function of the canonical eigenvalues and those which act on a complete covariance or correlation matrix.

#### The framework

Consider a simple 'animal model' for *q *traits, **y **= **Xb **+ **Zg **+ **e **with **y**, **b**, **g **and **e **the vectors of observations, fixed effects, additive genetic and residual effects, respectively, and **X **and **Z **the corresponding incidence matrices. Let **Σ***_G _*and **Σ***_E _*denote the matrices of additive genetic and residual covariances among the *q *traits. This gives a vector of parameters to be estimated, ***θ ***of length *q*(*q *+ 1), comprising the distinct elements of **Σ***_G _*and **Σ***_E_*. Furthermore, let Var (**g**) = **Σ***_G _*⊗ **A **= **G**, where **A **is the numerator relationship matrix between individuals, and $\text{Var}\;\left(e\right)=R=\sum_{k}^{+}R_{k}$, where 'Σ^+^' is the direct matrix sum. **R***_k _*is a function of **Σ***_E_*, e.g. for single records per trait it is the sub-matrix of **Σ***_E _*corresponding to the traits recorded for the *k*-th individual. The phenotypic covariance matrix of the vector of observations is then Var (**y**) = **ZGZ**' + **R **= **V**, and the pertaining REML log likelihood is, apart from a constant,

(2) $$\begin{array}{ll} \log L\left(\theta \right)= & -\frac{1}{2}\left(\log|V|+\log|X_{0}^{\prime }V^{-1}X_{0}|\right)\;\\ & +\left({\left(y-Xb\right)}^{\prime }V^{-1}\left(y-Xb\right)\right)\;\\ & \; \end{array}$$

where **X**_0 _is a full-rank submatrix of **X**, e.g. [5]. Regularized estimates can be obtained by maximizing the *penalized *likelihood

(3) $$\log L_{P}\left(\theta \right)=\log L\left(\theta \right)-\frac{1}{2}\psi P\left(\theta \right)$$

where the penalty P(θ) is a selected function of the parameters, aimed at reducing loss in their estimates, and *ψ *is a tuning factor which specifies the relative emphasis to be given to the penalty compared to the unpenalized estimator. For *ψ *= 0, this simplifies to the standard, unpenalized likelihood. The factor of 1/2 in (Eq. 3) is for algebraic consistency and could be omitted.

#### Penalties on eigenvalues

Recognition of the systematic bias in the eigenvalues of estimates of covariance matrices has led to the development of various estimators, which modify the eigenvalues whilst retaining the corresponding eigenvectors. As the mean eigenvalue is expected to be unbiased, a specific proposal has been to regress eigenvalues towards their mean to reduce their excessive spread.

Hayes and Hill [14] proposed to apply this type of shrinkage to the canonical eigenvalues (*λ_i_*), i.e. the eigenvalues of $\Sigma_{p}^{-1}\Sigma_{G},$ with **Σ***_p _*= **Σ***_G _*+ **Σ***_E _*the phenotypic covariance matrix. The equivalent to such 'bending' in a (RE)ML framework is obtained by placing a penalty proportional to the variance among the canonical eigenvalues on the likelihood [15]:

(4) $$P_{\lambda }\propto \text{tr}{\left(\Lambda -\bar{\lambda }I\right)}^{2}\;\mathrm{with}\;\bar{\lambda }=\text{tr}(\Lambda )/q$$

for **Λ **= Diag {*λ_i_*}. The canonical decomposition gives **Σ***_G _*= **TΛT**' and **Σ**_E _= **T**(**I - Λ**)**T**', with **I **an identity matrix and **T **the matrix of eigenvectors of $\Sigma_{p}^{-1}\Sigma_{G},$ scaled by a matrix square root of **Σ***_p_*. Hence, $P_{\lambda }$ penalizes both **Σ***_G _*and **Σ***_E _*at the same time. Thus, while the motivation for 'bending' appears somewhat *ad hoc*, the same penalty can be derived assuming the canonical eigenvalues have a Normal prior [10].

Penalizing eigenvalues transformed to logarithmic scale, i.e. defining **Λ **= Diag{log(*λ_i_*)}, yields a related penalty, $P_{\lambda }^{\ell },$ similar to the log eigenvalue posterior mean shrinkage estimator suggested by Daniels and Kass [19]. While quadratic penalties on (1 - *λ_i_*) and *λ_i _*are equivalent, this does not hold on the log scale. Hence, for **Λ**_1 _= Diag{log(*λ_i_*)} and **Λ**_2 _= Diag{log(1 - *λ_i_*)} (with ${\bar{\lambda }}_{i}=\text{tr(}\Lambda_{i})∕q),$ a third penalty is

(5) $$P_{\lambda }^{\ell 2}\propto \text{tr}{\left(\Lambda_{1}-{\bar{\lambda }}_{1}I\right)}^{2}+\text{tr}{\left(\Lambda_{2}-{\bar{\lambda }}_{2}I\right)}^{2}$$

For **Σ***_G _*positive semi-definite, the canonical eigenvalues lie in the interval [0,1]. Hence a natural alternative to a normal prior is the beta distribution, which is usually defined on this domain and is thus frequently used as prior for binomial proportions in a Bayesian setting. It has two shape parameters, *α *> 0 and *β *> 0, and probability density function

(6) $$p\left(x\right)=\frac{\Gamma \left(\alpha +\beta \right)}{\Gamma \left(\alpha \right)\Gamma \left(\beta \right)}x^{\alpha -1}{\left(1-x\right)}^{\beta -1}$$

with Γ(·) denoting the gamma function, and mean *α*/(*α *+ *β*). Hence, for *α *= *β*, *p*(*x*) is symmetric with the mean at 0.5. For *α *> 1 and *β *> 1, it is uni-modal with probability mass increasingly concentrated at the mean as *α *and *β *increase. A restricted domain [*x*_1_, *x*_2_] (with *x*_1 _and *x*_2 _the lower and upper limits for *x*) can be taken into account by fitting a four parameter beta function [20] or by replacing *x *in (Eq. 6) with x^* ^= (*x - x*_1_)/(*x*_2 _- *x*_1_). The distribution of estimates of the canonical eigenvalues clearly depends on the population parameters and may well not cover the whole interval [0,1]. As we expect standard estimates of eigenvalues to be over-dispersed, a suitable, if somewhat inflated, estimate of the range may be given by the estimates of the extreme values from an unpenalized analysis (i.e. *ψ *= 0), denoted henceforth by a superscript of 0. Assuming eigenvalues are numbered in descending order of magnitude, this gives $\lambda_{i}^{⋆}=\left(\lambda_{i}-\lambda_{q}^{0}\right)∕\left(\lambda_{1}^{0}-\lambda_{q}^{0}\right)$ and penalty

(7) $$P_{\beta }^{a}\propto \left(\alpha -1\right)\log\left(\lambda_{i}^{⋆}\right)+\left(\beta -1\right)\log\left(1-\lambda_{i}^{⋆}\right)$$

A suitable choice for the shape parameters might be *α *= *β *= 2, 3,..., i.e. a symmetric distribution for $\lambda_{i}^{*}$ with probability mass somewhat more spread out than a normal distribution.

Alternatively, *α *and *β *can be estimated from estimates $\lambda_{i}^{0}$. Using the fact that the mean and variance of the standard beta distribution are *α/*(*α *+ *β*) and *αβ*(*α *+ *β*)^-2^(*α *+ *β *+ 1)^-1^, results in the method of moment estimators $\alpha =\bar{\lambda }v$ and $\beta =\left(1-\bar{\lambda }\right)v$, with $v=q\bar{\lambda }(1-\bar{\lambda })/\sum_{i=1}^{q}{\left(\lambda_{i}^{0}-\bar{\lambda }\right)}^{2})-1$ and $\bar{\lambda }$ the mean of the $\lambda_{i}^{0}$[20]. This may result in estimates of *α *and *β *less than unity, implying probability distributions that are U- or J-shaped with a high mass at the extremes. To ensure a uni-modal beta distribution, we add a constant *z *(*z *≥ 0). This gives penalty

(8) $$P_{\beta }^{b}\propto \left(\alpha +z-1\right)\log\left(\lambda_{i}\right)+\left(\beta +z-1\right)\log\left(1-\lambda_{i}\right)$$

Penalties considered so far implied that estimated eigenvalues were samples from a distribution with a common mean $\bar{\lambda }$. However, while quadratic penalties on eigenvalues or eigenvalues transformed to logarithmic scale have been found to be highly effective when the corresponding population values are similar, they resulted in substantial over-shrinkage when population values were spread apart [3,15,19]. Hence, if population eigenvalues are markedly different, it may be advantageous to shrink towards individual targets. Ordering variables according to size introduces a specific distribution. The *i*-th order statistic of a *q*-variate sample is the *i*-th smallest value. Assuming a uniform distribution, the order statistics on the unit interval have marginal beta distributions with scale parameters *i *and *q *- *i *+ 1. Treating values $\lambda_{i}^{⋆}$ as independent order statistics gives the penalty

(9) $$P_{\beta }^{c}\propto \overset{q}{\underset{i=1}{\sum }}\left(z+i-1\right)\log\left(\lambda_{i}^{⋆}\right)+\left(z+q-i\right)\log\left(1-\lambda_{i}^{⋆}\right)$$

Again, we allow for a modifying constant *z *in (Eq. 9). For the distribution of order statistics, *z *= 0. A value of *z *> 0 causes individual distributions to be 'squashed' together, i.e. yields a compromise between the assumption of a common mean for the $\lambda_{i}^{⋆}$ and that of an even distribution over the unit interval.

#### Penalties on matrix divergence

Motivated by the historical emphasis on the role of sample eigenvalues of covariance matrices, we have concentrated on penalties on these characteristics so far. A conceptually simpler alternative is to consider the covariance matrix as a whole and its prior distribution.

A standard assumption in Bayesian estimation of covariance matrices is that of an inverse Wishart prior distribution, because, for observations with a multivariate normal distribution, this is a conjugate prior. It has the probability density function $p\left(\Sigma |\Omega ,v\right)\propto |\Sigma {|}^{\frac{1}{2}\left(v+q+1\right)}\exp\left[-\frac{1}{2}\;\text{tr}\left(\Sigma^{-1}\Omega \right)\right]$ e.g. [21], with **Ω **denoting the scale parameter and *v *the degree of belief we assign to the prior. Omitting terms not depending on **Σ **or **Ω **and taking logarithms gives (*v *+ *q *+ 1) log |**Σ**| + *v *tr(**Σ**^-1 ^Ω). Corresponding to the penalties that 'borrow strength' from the phenotypic covariance matrix considered above, a penalty which regularizes the estimate of **Σ***_G _*by shrinking it towards **Σ***_p _*can be obtained by using **Σ***_p _*as a scale matrix. Adopting an empirical Bayes approach, we substitute the estimate from an unpenalized REML analysis, $\Sigma_{P}^{0}$, in place of **Σ***_P _*[22]. Further, replacing *v *with the tuning factor *ψ*, then gives a penalty

(10) $$P_{\Sigma }\propto C\log|\Sigma_{G}|+\;\text{tr}\left(\Sigma_{G}^{-1}\Sigma_{P}^{0}\right)$$

with *C *= (*ψ *+ *q *+ 1)/*ψ*. If *C *is approximated with unity, $P_{\Sigma }$ is proportional to the Kullback-Leibler divergence between **Σ***_G _*and $\Sigma_{P}^{0}$, which is the entropy loss *L*_1_(·) with **Σ **and $\hat{\Sigma }$ exchanged [23]. The relationship between $P_{\Sigma }$ and $P_{\lambda }$ can be seen by rewriting (Eq. 10) in terms of the canonical decomposition, which gives $P_{\Sigma }\propto C\left(\log|\Lambda |+\log|T\;T\prime |\right)+\text{tr}\left(\Lambda^{-1}T^{-1}\Sigma_{P}^{0}T^{-T}\right)$. Assuming that $\Sigma_{P}^{0}\approx {T\;T}^{\prime }$, i.e. that the estimate of the transformation and of the phenotypic covariance matrix are largely unaffected by penalized estimation, gives $P_{\Sigma }\propto C\log|\Lambda |+\;\text{tr}\left(\Lambda^{-1}\right)\propto \sum_{i}^{q}C\log\left(\lambda_{i}\right)+\lambda_{i}^{-1}$. This shows that $P_{\Sigma }$ implies a substantial penalty on the smallest canonical eigenvalues. We can also penalize both **Σ***_G _*and **Σ***_E _*simultaneously using

(11) PΣ2∝CG log∣ΣG∣+tr(ΣG-1ΣP0)+CE log∣ΣE∣+tr(ΣE-1ΣP0)

weighted by either a joint (*C_G _*= *C_E_*) or separate tuning factors.

Based on empirical evidence that estimates of genetic (*r_G _*) and phenotypic (*r_P_*) correlations are often similar, Cheverud [24] proposed to substitute *r_P _*for *r_G _*if the data did not support accurate estimation of *r_G_*. Adopting this suggestion, Meyer and Kirkpatrick [25] demonstrated that estimating **Σ***_G _*and **Σ***_E _*or **Σ***_P _*under the assumption of a joint correlation structure resulted in highly parsimonious models and a dramatic reduction in mean square errors when the underlying assumptions were approximately true. Conversely, estimates could be substantially biased if they were not. A more flexible alternative is to penalize the divergence between estimates of the genetic (**R***_G_*) and phenotypic correlation (**R***_P_*) matrix, i.e. to shrink the estimate of **R***_G _*towards $R_{P}^{0}$. Analogous to (Eq. 10), this can be achieved by using a penalty

(12) $$P_{\rho }\propto C\log|R_{G}|+\;\text{tr}\left(R_{G}^{-1}R_{P}^{0}\right)$$

or

(13) Pρ2∝CG log∣RG∣+tr(RG-1RP0)+CE log∣RE∣+tr(RE-1RP0)

More generally, such penalty on the complete matrix can be used to shrink an estimated covariance (or correlation) matrix towards any chosen structure. This allows for a data-driven compromise between the assumed structure and an unstructured matrix. For instance, Chen [26] presented an empirical Bayesian approach to estimate a covariance matrix by shrinking towards a prior that was assumed to have a factor-analytic or compound symmetric structure. More recently, Schäfer and Strimmer [27] considered shrinkage towards a number of target matrices with diagonal structure or constant correlations. Within our penalized (RE)ML framework, this can be achieved by substituting the structured matrix for the scale matrix **Ω **in (Eq. 10). This may be a suitable matrix chosen *a priori *or, in an empirical vein, an unpenalized estimate obtained from the data, imposing the structure selected.

## Simulation study

### Simulation set-up

Data for a simple paternal half-sib design comprising *s *unrelated sires with *n *= 10 progeny each were simulated by sampling from appropriate multivariate normal distributions for *q *= 5 and *q *= 9 traits. Sample sizes considered were *s *= 50, 100, 150, 200, 300, 400, 600 and 1000. A total of 90 sets of population parameters, 60 for *q *= 5 and 30 for *q *= 9 traits were examined.

Population parameters for *q *= 5 were obtained by combining 12 sets of heritabilities (A to L) with five scenarios for genetic (*r_G_*) and residual (*r_E_*) correlations and phenotypic variances, named *I *to V. This resulted in 60 combinations, labelled A-*I *to L-*V *in the following. Similarly, 10 sets of heritabilities (M to V) for *q *= 9 traits were combined with correlation scenarios *I*, *VI *and *VII *to yield combinations M-*I *to V-*VII*. Heritabilities were chosen so that the mean was 0.4 (A to G and M to Q), 0.3 (H) or 0.2 (I to L and S to V), with values declining with an increasing trait number. There were different degrees of spread in heritabilities, ranging from equal values for all traits (A, I, M and R) to sets of values which spanned a length interval of 0.80 (E, H, and O) and sets with a very uneven distribution of heritabilities (G, H, L, U and V). Sets of population values for the correlations that were used were *r_Gij _*= *r_Eij _*= 0, *r_Gij _*= 0.8 and *r_Eij _*= 0, *r_Gij _*= 0.6^|*i*-*j*| ^and *r_Eij _*= -0.4^|*i-j*| ^+ 0.5, *r_Gij _*= -0.8^|*i*-*j*| ^+ 0.02*i *and *r_Eij _*= -0.4^|*i*-*j*| ^+ 0.5, *r_Gij _*= -1*^i^*0.05*j *+ 0.5 and *r_Eij _*= -1*^j^*0.1*i *+ 0.2, *r_Gij _*= 0.7^|*i*-*j*| ^and *r_Eij _*= -1*^j^*0.05*i *+ 0.2, and *r_Gij _*= -0.8^|*i*-*j*| ^+ 0.02*i *and *r_Eij _*= -0.2^|*i*-*j*| ^+ 0.5, for correlation scenarios *I *to *VII*, respectively. Population phenotypic variances were $\sigma_{i}^{2}=1$ for *I*, $\sigma_{i}^{2}=1.5^{i-1}$ for *II*, $\sigma_{1}^{2}=\sigma_{5}^{2}=3,\;\sigma_{2}^{2}=\sigma_{4}^{2}=2$ and $\sigma_{3}^{2}=1$ for *I II*, *IV *and *V*, and $\sigma_{1}^{2}=\sigma_{4}^{2}=\sigma_{6}^{2}=\sigma_{9}^{2}=2,\;\sigma_{2}^{2}=\sigma_{5}^{2}=\sigma_{8}^{2}=1$ and $\sigma_{3}^{2}=\sigma_{7}^{2}=3$ for *VI *and *VII*. This yielded coefficients of variation among the corresponding canonical eigenvalues ranging from 0 to 175%. A total of 1000 replicates per case and sample size were sampled.

### Analyses

REML estimates of **Σ***_G _*and **Σ***_E _*for each sample were obtained for different penalties and tuning factors by using a method of scoring algorithm to locate the maximum of log L(θ) or log $L_{p}\left(\theta \right)$, followed by simple derivative-free search steps to ensure that convergence had been reached. This was done using a parameterisation to the elements of the canonical decomposition, *λ_i _*and *t_ij _*∈ **T**, as described by Meyer and Kirkpatrick [15], restraining estimates of *λ_i _*to the interval of [0.0001, 0.9999].

A total of 10 penalties were examined, six penalties on the canonical eigenvalues, $P_{\lambda },P_{\lambda }^{\ell },P_{\lambda }^{\ell 2},P_{\beta }^{a}$ for $\alpha =\beta =2,\;P_{\beta }^{b}$ for *z *= 1 and $P_{\beta }^{c}$ for *z *= 1, and four penalties on matrices, $P_{\Sigma },\;P_{\Sigma }^{2},P_{\rho }^{}$ and $P_{\rho }^{2}$, as described above. All employed a single tuning factor. In addition, two different tuning factor to the parts of penalties $P_{\lambda }^{\ell 2},\;P_{\Sigma }^{2}$ and $P_{\rho }^{2}$ that corresponded to genetic and residual components were employed.

### Estimating the tuning factor

To determine the tuning factor (*ψ*) for each analysis, estimates of **Σ***_G _*and **Σ***_E_*, denoted as $\Sigma_{G}^{\psi }$ and $\Sigma_{E}^{\psi }$, were obtained for a range of possible values for *ψ*. A total of 311 values were used, comprising values of 0 to 2 in steps of 0.1, 2.2 to 5 in steps of 0.2, 5.5 to 10 in steps of 0.5, 11 to 100 in steps of 1, 102 to 250 in steps of 2, 255 to 500 in steps of 5 and 510 to 1000 in steps of 10. The 'best' value was then chosen using three different approaches.

First, as in previous work [15], knowledge of the population parameters was used. For each *ψ *and estimates $\Sigma_{G}^{\psi }$ and $\Sigma_{E}^{\psi }$, the corresponding *unpenalized *log likelihood was calculated as

(14) $$\begin{array}{ll} \log L{\left(\theta \right)}^{\psi }= & -\frac{1}{2}\left[\left(s-1\right)\left(\log|\Sigma_{B}|+\text{tr}\left(\Sigma_{B}^{-1}M_{B}\right)\right)\right)\;\\ & +\left(s\left(n-1\right)\left(\log|\Sigma_{W}|+\text{tr}\left(\Sigma_{W}^{-1}M_{W}\right)\right)\right]\;\\ & \; \end{array}$$

with $\Sigma_{W}=\Sigma_{E}^{\psi }+\frac{3}{4}\Sigma_{G}^{\psi }$ and $\Sigma_{B}=\Sigma_{W}+\frac{1}{4}n\Sigma_{G}^{\psi }$. This requires validation 'data' which, for a paternal half-sib design, can be summarized as the matrices of mean squares and cross-products between (**M***_B_*) and within (**M***_W_*) sires, as from an analysis of variance. For strategy V1, **M***_B _*and **M***_W _*were obtained by sampling one additional data set from the same distribution as the data used in the analysis. For strategy V∞, **M***_B _*and **M***_W _*were constructed from the population parameters. This is equivalent to sampling an infinite number of additional data sets, hence the notation V∞. For both strategies, the value of *ψ *which maximised $\log L{\left(\theta \right)}^{\psi }$ was then chosen as the appropriate tuning factor.

Secondly, *K*-fold cross-validation (CV) was used to estimate *ψ *using only the data available. For this, data were split into *K *folds of approximately equal size by sequentially assigning complete sire families to subsets. For *i *= 1, *K*, the *i*-th subset was set aside for validation, while all the remaining *K*-1 subsets were used to obtain estimates $\Sigma_{G}^{\psi }$ and $\Sigma_{E}^{\psi }$ for all values of *ψ *considered. Corresponding values for the unpenalized likelihood, $\log L{\left(\theta \right)}_{i}^{\psi }$ (Eq. 14), in the validation data were then obtained and accumulated across folds. Finally, *ψ *was chosen as the value for which the average likelihood, $\sum_{i=1}^{K}\log L{\left(\theta \right)}_{i}^{\psi }∕K$, was maximized. Values of *K *= 3 and 5 were considered, with the corresponding strategies denoted as CV3 and CV5 in the following.

The third approach used simply involved choosing *ψ *as the largest value of *ψ *for which the reduction in the unpenalized likelihood due to penalization from the maximum at $\psi =0,\;|\log L{\left(\theta \right)}^{\psi }-\log L{\left(\theta \right)}^{0}|$, did not exceed a selected value. The limit chosen was the $\chi_{\gamma }^{2}$ value $\left(\times \frac{1}{2}\right)$ employed in a likelihood ratio test of a single parameter with error probability *γ*, 1.92 for *γ *= 0.05. This will be referred to as strategy L5%.

### Summary statistics

As suggested by Lin and Perlman [28], the effect of penalized estimation was evaluated as the percentage reduction in average loss (PRIAL) due to penalization,

$$100\left[{\bar{L}}_{1}\left(\Sigma_{X},\;\Sigma_{X}^{0}\right)-{\bar{L}}_{1}\left(\Sigma_{X},\;\Sigma_{X}^{\psi }\right)\right]∕{\bar{L}}_{1}\left(\Sigma_{X},\;\Sigma_{X}^{0}\right)$$

where $\Sigma_{X}^{0}$ is the standard, unpenalized REML estimate of **Σ***_X _*and $\Sigma_{X}^{\psi }$ the penalized estimate, for *X *= *G*, *E *and *P*, and ${\bar{L}}_{1}\left(\cdot \right)$ denotes the entropy loss (see (Eq. 1)), averaged over replicates. In addition, the absolute and relative bias (in %) for parameter *θ_i _*were calculated as $|{\hat{\theta }}_{i}-\theta_{i}|$ and 100 $\left({\hat{\theta }}_{i}-\theta_{i}\right)∕\theta_{i}$, respectively.

## Results

### Comparing penalties

Mean PRIAL values across all cases for individual covariance matrices and all penalties considered are summarized in Table 1 for a sample size of *s *= 100. Using known population values (strategy V∞), achieved reductions in average loss in estimates of **Σ***_G _*were substantial, ranging from about 60% to more than 72%. The main exception was $P_{\lambda }$ (which penalized the canonical eigenvalues rather than their logarithmic values), for which PRIALs for **Σ***_E _*were substantially higher than for **Σ***_G_*. On average PRIAL values were somewhat smaller for *q *= 9 than *q *= 5 traits because cases for *q *= 9 comprised more unfavourable scenarios, i.e. population values with a large and uneven spread of the canonical eigenvalues.

**Table 1:** Mean percentage reduction in average loss in estimates of covariance matrices (**Σ***_G_*: genetic, **Σ***_E_*: residual and **Σ***_p_*: phenotypic).

Cov.^1^	Strategy^2^	Penalty									
		
		$P_{\lambda }$	$P_{\lambda }^{\ell }$	$P_{\lambda }^{\ell 2}$	$P_{\beta }^{a}$	$P_{\beta }^{b}$	$P_{\beta }^{c}$	$P_{\Sigma }$	$P_{\Sigma }^{2}$	$P_{\rho }$	$P_{\rho }^{2}$
**5 traits**											
**Σ***_G_*	V∞	35.8	71.3	72.9	66.7	71.4	67.9	70.6	70.0	72.0	72.2
	CV3	23.1	55.9	60.7	59.2	58.1	61.1	54.9	52.9	54.4	56.9
	L5%	41.3	68.3	70.2	67.6	69.5	69.3	64.1	66.7	70.5	71.5
**Σ***_E_*	V∞	57.9	43.4	61.6	59.3	60.9	59.7	13.3	54.2	37.3	60.0
	CV3	14.1	26.7	44.3	38.7	36.0	39.6	10.7	43.0	22.8	40.9
	L5%	43.6	35.0	55.9	54.2	54.1	54.0	7.2	51.4	33.2	55.7
**Σ***_P_*	V∞	1.1	1.2	1.3	1.3	1.2	1.2	1.2	1.7	2.2	2.4
	CV3	-0.4	0.4	0.5	0.3	0.1	0.3	0.2	0.1	0.4	0.8
	L5%	-0.7	0.7	0.8	0.5	0.5	0.5	0.3	1.0	1.0	1.2
**9 traits**											
**Σ***_G_*	V∞	48.4	64.8	68.4	65.3	68.9	66.7	64.0	62.8	71.3	73.3
	L5%	24.1	67.5	67.7	65.4	66.5	66.4	68.0	67.7	69.5	69.4
**Σ***_E_*	V∞	62.9	60.5	68.8	67.8	67.3	68.3	10.4	61.1	57.9	70.2
	L5%	63.0	16.4	59.3	60.9	62.6	61.7	9.9	47.4	17.2	56.3
**Σ***_P_*	V∞	1.3	1.9	1.9	2.0	1.8	2.0	1.2	1.7	2.5	3.0
	L5%	1.2	0.5	1.1	1.2	1.3	1.2	0.6	0.7	1.1	1.2

Data for *s *= 100 sire families; using different penalties and up to three strategies to determine the tuning factor (V∞: using population values, CV3: using 3-fold cross-validation, and L5%: limiting the change in log likelihood)

^1^Covariance matrix

^2^Method to determine the tuning factors

As reported earlier [15], taking logarithms of the canonical eigenvalues $\left(P_{\lambda }^{\ell }\right)$ greatly improved the efficacy of a penalty proportional to their squared deviations from the mean. Because canonical eigenvalues are a function of both **Σ***_G _*and **Σ***_E_*, all penalties on *λ_i _*yielded marked improvements in estimates of **Σ***_E _*as well as **Σ***_G_*. Considering log(1 - *λ_i_*) in addition to log(*λ_i_*) ($P_{\lambda }^{\ell 2}$ and all $P_{\beta }$) increased PRIALs for **Σ***_E _*further without affecting estimates of **Σ***_G _*detrimentally. Among the penalties based on the beta distribution, those that estimated the scale parameters $\left(P_{\beta }^{b}\right)$ performed best. With different underlying assumptions, the similarity of results for $P_{\beta }^{c}$, the penalty based on order statistics, and results for penalties that assumed a common mean of all *λ_i _*was somewhat surprising.

Whilst achieving comparable PRIAL on **Σ***_G_*, penalizing the difference between genetic and phenotypic covariance or correlation matrices behaved different to penalties on canonical eigenvalues (Table 1). As to be expected, considering **Σ***_G _*only $\left(P_{\Sigma }\right)$ yielded only small improvements in estimates of **Σ***_E_*. Adding a corresponding penalty on the residual covariances $\left(P_{\Sigma }^{2}\right)$ increased PRIAL for **Σ***_E _*to levels comparable to those obtained when penalizing canonical eigenvalues, again without reducing the mean PRIAL for **Σ***_G _*notably. For *q *= 9 traits, there was an unexpected but substantial difference between imposing penalties on the covariance versus the correlation matrix. Penalizing both genetic and residual correlations increased the PRIAL for ${\hat{\Sigma }}_{G}$ by 2% ($P_{\rho }^{2}$*vs. *$P_{\rho }$). In contrast, corresponding differences for *q *= 5 were considerably smaller. It is not clear how much this was an effect of the dimension or due to differences in population values. Allowing for different tuning factors for parts of the penalty that correspond to genetic and residual effects increased the PRIAL for **Σ***_G _*for *q *= 5 from 72.9 to 73.7% for $P_{\lambda }^{\ell 2}$, from 70.0 to 72.7% for $P_{\Sigma }^{2}$ and from 72.2 to 74.3% for $P_{\rho }^{2}$, i.e. by less than 3%. Corresponding PRIAL for **Σ***_E _*were 65.6% $\left(P_{\lambda }^{\ell 2}\right)$, 64.9% $\left(P_{\Sigma }^{2}\right)$ and 62.7%, i.e. increased by more than 10% for $P_{\Sigma }^{2}$. While non-negligible, the gains for estimates of **Σ***_G _*were deemed too small to off-set the dramatically increased computational requirements that arose from the two-dimensional search for the optimal tuning factors, and was not given further consideration.

**Figure 1:**
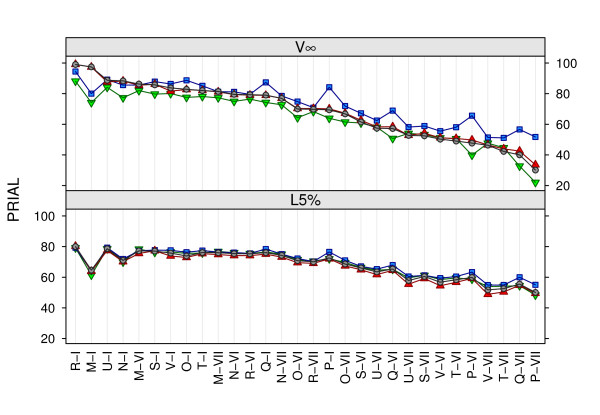
**Percentage reduction in average loss (PRIAL) in estimates of the genetic covariance matrix for individual cases and different penalties**. Data for *q *= 9 traits, determining tuning factors on the basis of population values (V∞) and by limiting the change in likelihood (L5%); ▼ $P_{\Sigma }$, ■ $P_{\rho }^{2}$, ▲ $P_{\beta }^{b}$ and ● $P_{\lambda }^{\ell 2}$, see text of acronyms

Mean PRIAL values discussed so far concealed a considerable range and variation in the ranking of penalties for individual cases. This is illustrated in Figure 1, which shows the PRIAL for **Σ***_G _*for *q *= 9 traits, with individual cases in declining order of the PRIAL obtained using $P_{\lambda }^{\ell 2}$. For strategy V∞, penalties on canonical eigenvalues that assumed a common mean performed best when populations values for the *λ_i _*were fairly similar, e.g. for R-*I *and M-*I *all population values were equal. For *q *= 9, there was little difference in PRIAL for **Σ***_G _*between penalties that assumed a normal distribution on the logarithmic scale ($P_{\lambda }^{\ell }$ and $P_{\lambda }^{\ell 2}$) or a beta distribution with estimated scale parameters $\left(P_{\beta }^{b}\right)$, although some tendency for $P_{\beta }^{b}$ to yield slightly higher values for cases where penalized estimation worked least well was evident. Conversely, penalties derived assuming an inverse Wishart matrix prior mostly yielded larger PRIAL for the other cases, in particular when penalizing the difference between genetic and phenotypic correlations. For *q *= 5, penalties $P_{\rho }$ and $P_{\rho }^{2}$ performed best for 35% of the individual cases considered, mainly those for which PRIAL for **Σ***_G _*were less than average, while $P_{\lambda }^{\ell }$ and $P_{\lambda }^{\ell }$ yielded the highest values for 37% of cases. For *q *= 9, $P_{\rho }^{2}$ yielded the highest PRIAL for 80% of cases - mostly due to population canonical eigenvalues having a substantial spread for the majority of these cases.

### Estimating tuning factors

A crucial part of penalized estimation is the estimation of the appropriate tuning factor to be used. Mean PRIAL values for **Σ***_G _*for different strategies to determine *ψ *are summarized in Table 2 for selected penalties, *q *= 5 traits and *s *= 100 sires, together with the average proportion of replicates for which penalization increased rather than decreased the loss in **Σ***_G_*. Corresponding PRIAL values for all penalties for strategies V∞, CV3 and L5% are given in Table 1. Clearly, mean values well above 70% when using the population values (V∞) present an overly optimistic view of the efficacy of penalized estimation. Considering only one additional sample for validation (strategy V1) introduced considerable sampling error and thus reduced PRIAL achieved by about 10%.

**Table 2:** Mean percentage reduction in average loss for the genetic covariance matrix together with the average proportion of replicates for which penalisation increased loss

Penalty	% reduction in average loss					% replicates with increased loss				
		
	V∞	V1	CV3	CV5	L5%	V∞	V1	CV3	CV5	L5%
$P_{\lambda }^{\ell }$	71.3	60.6	55.9	50.4	68.3	7.3	8.7	14.6	14.6	12.0
$P_{\lambda }^{\ell 2}$	72.9	63.7	60.7	58.1	70.2	6.5	7.5	13.0	13.2	10.0
$P_{\beta }^{b}$	71.4	62.9	58.1	53.9	69.5	6.4	7.5	13.6	14.0	9.8
$P_{\Sigma }$	70.6	60.6	54.9	52.7	64.1	4.6	8.9	15.4	15.5	15.6
$P_{\rho }$	72.0	62.9	54.4	51.6	70.5	4.0	7.1	9.9	10.2	9.2

Data for *q *= 5 traits and *s *= 100 sire families; using different penalties and strategies to determine the tuning factor (V∞: using population values, V1: sampling one additional data set, CV3 and CV5: 3- and 5- fold cross-validation, and L5%: limiting the change in log likelihood)

Examining regularized estimation of a single covariance matrix, Rothman et al. [29] reported that strategy V1 yielded similar results to CV. However, in our case, mean PRIAL values using CV to determine *ψ *were consistently lower, i.e. suffered from additional noise (Table 2). Somewhat surprisingly, PRIAL tended to decrease with the number of folds considered, *K*. This was accompanied by increasing variability of results for individual cases. Clearly, there was a trade-off between the sizes of the training and validation sets. One might expect that a smaller training set (low *K*) would yield a *ψ *that was too high, as it pertained to the sample size of the subset, while a larger number of folds (high *K*) might off-set potential inabilities to ascertain optimal values for *ψ *due to the limited size of the validation set. However, results for CV5 were consistently worse than for CV3. Additional analyses for *K *= 10 (not shown) yielded even lower PRIAL than CV5. Inspection of the mean tuning factors $\left(\bar{\psi }\right)$ did reveal a trend for $\bar{\psi }$ to decline with increasing *K*. For penalties $P_{\beta }^{b},\;P_{\Sigma }$ and $P_{\rho }$, values for $\bar{\psi }$ from CV were substantially higher than for strategy V∞, suggesting that lower PRIALs from CV were due to over-penalization. For $P_{\lambda }^{\ell }$ and $P_{\lambda }^{\ell 2}$, results were less consistent: for these penalties *ψ *determined using V∞ tended to be very high for cases with little spread in the population *λ_i_*, while corresponding values using CV tended to be substantially lower, so that the average, $\bar{\psi }$, from strategies V∞, CV3 and CV5 were similar. CV also reduced differences between penalties. Interestingly, penalty $P_{\beta }^{c}$ appeared least affected by the 'noise' introduced by estimating *ψ*. For strategy CV3, $P_{\beta }^{c}$ yielded the highest PRIAL in **Σ***_G _*for 35% of the individual cases (*q *= 5 and *s *= 100), compared to 2% for strategy V∞.

Difficulties in deriving the optimal 'bending' factor theoretically, led Hayes and Hill [14] to suggest a choice based on sample size. An alternative in a likelihood framework is to select the tuning factor so that the corresponding reduction in the unpenalized likelihood does not exceed a given limit. When carrying out a likelihood ratio test for the difference between estimates from different models, minus twice the difference in log likelihood is contrasted to a value of the *χ*^2 ^distribution corresponding to the number of parameters tested and an error probability *γ*. The smallest number of parameters which can be tested is *p *= 1. Hence, choosing *ψ *as the largest value for which the resulting change in log L(θ) (sign ignored) does not exceed $\frac{1}{2}\chi_{\gamma }^{2}$ for one degree of freedom will result in a statistically non-significant change in estimates. While it may not yield the optimal amount of regularization, it allows for selection of a mild degree of penalization without having to justify significant changes in parameter estimates. In addition, computational requirements for such a strategy are considerably less than for CV.

As shown in Table 1 and Table 2, determining *ψ *in this way yielded substantially improved estimates of **Σ***_G_*, with PRIAL consistently higher than for CV. Values for the average tuning factor $\bar{\psi }$ (not shown) were markedly and consistently lower than those for V∞, indicating that this approach indeed resulted in under-penalization. This held especially for cases with similar population canonical eigenvalues (E-*I*, H-*I*, I-*I*, M-*I *and R-*I*). As illustrated in Figure 1, choosing *ψ *using this strategy also blurred differences between penalties. In a number of cases, in particular for *q *= 9 traits, PRIAL for **Σ***_G _*from strategy L5% were higher than those from V∞.

### Effects of sample size

**Figure 2:**
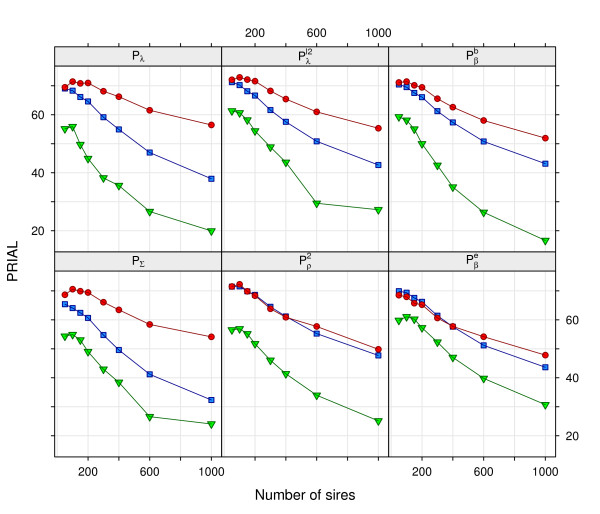
**Mean percentage reduction in average loss (PRIAL) in estimates of the genetic covariance matrix for different sample sizes**. Data for *q *= 5 traits; ● using population values (V∞), ■ limiting the change in likelihood (L5%) and ▼ using cross-validation (CV3) to determine the tuning factors

The effect of sample size on the efficacy of regularized estimation is illustrated in Figure 2 for *q *= 5. Clearly, penalization was most advantageous for small samples, with mean PRIAL for **Σ***_G _*decreasing substantially as the number of sire families increased. There were marked differences between penalties and strategies to determine *ψ*, especially in the rate of decline of PRIAL with increasing *s*. This rate was least for penalty $P_{\rho }^{2}$ and, moreover, choosing tuning factors on the basis of the change in log L(θ) performed almost as well as exploiting knowledge of the population values. Using $P_{\rho }^{2}$ resulted in the highest PRIAL for both **Σ***_G _*and **Σ***_E _*for all sample sizes, when using the change in log L(θ) to estimate *ψ*.

As noted above, PRIAL in **Σ***_G _*when using CV to determine the tuning factor were substantially less than for the other strategies. This difference tended to increase with sample size. Whilst consistently performing worst for strategy V∞, the penalty on *λ_i _*derived from the distribution of order statistics $\left(P_{\beta }^{c}\right)$ resulted in the highest PRIAL in **Σ***_G _*for strategy CV3. It is not clear what this comparatively larger robustness against noise in estimates of *ψ *can be attributed to. The decline in PRIAL with sample size was clearly a function of the number of traits considered, with reductions for *q *= 9 markedly smaller. For instance, for $P_{\rho }^{2}$ and strategy L5% the average PRIAL in **Σ***_G _*declined from 69.4% for *s *= 100 to 64.1% for *s *= 400 and 60.2% for *s *= 1000. Respective values for $P_{\lambda }^{\ell 2}$ were 67.7%, 64.2% and 54.2%. This suggests that mild penalization is advantageous even for larger samples as the dimensions of the covariance matrices to be estimated increase.

### Bias

**Figure 3:**
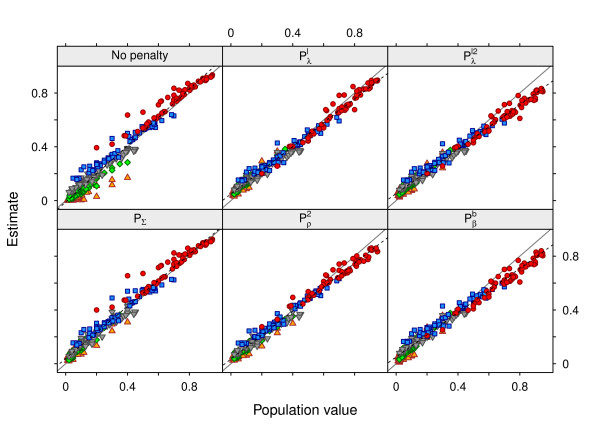
**Mean estimates of canonical eigenvalues for individual cases and different penalties**. Data for *q *= 5 traits and *s *= 100 sires, using population values (strategy V∞) to determine the tuning factor; ● first, ■ second, ▼ third, ♦ fourth and ▲ fifth eigenvalue

As emphasized earlier, regularized estimation entails a trade-off between sampling variance and bias. Table 3 gives the mean relative bias in estimates of canonical eigenvalues for a sample size of *s *= 100 sires and strategy V∞. Figure 3 further illustrates the relationship between estimates of *λ_i _*and their true values for selected penalties and strategy V∞, with the solid line showing a one-to-one correspondence (unbiased estimates) and the dashed line representing the linear regression of estimates on population values. Patterns obtained when selecting the tuning factor using L5% or CV were similar. As expected, without penalization, estimates of the largest values were biased upwards and those of the smallest values biased downwards. Whilst the mean was expected to be estimated without bias, a small upwards bias in the average eigenvalue, $\bar{\lambda }$, together with a clustering of the smallest *λ_i _*at zero were evident, reflecting the effects of constraints on the parameter space. A penalty on canonical eigenvalues tended to result in over-shrinkage, i.e. causing a downward bias of the largest and an upward bias of the smallest values. This was the more pronounced the further the population *λ_i _*were spread apart. Similar results for a single matrix were reported by Daniels and Kass [19]. While the relative bias was substantial for the smallest *λ_i_*, absolute changes tended to be small and penalization clustered estimates closer to the one-to-one line.

**Table 3:** Mean relative bias (in %) in estimates of the *i*-th canonical eigenvalue and of the mean eigenvalue $\left(\bar{\lambda }\right)$, and mean absolute bias in estimates of the *i*-th heritability (×100).

Penalty												
		
*q*^1^	*i*	None	$P_{\lambda }$	$P_{\lambda }^{\ell }$	$P_{\lambda }^{\ell 2}$	$P_{\beta }^{a}$	$P_{\beta }^{b}$	$P_{\beta }^{c}$	$P_{\Sigma }$	$P_{\Sigma }^{2}$	$P_{\rho }$	$P_{\rho }^{2}$
**Canonical eigenvalues**												
5	$\bar{\lambda }$	2.3	-5.4	6.6	2.1	3.4	-1.2	1.0	11.2	10.9	4.7	2.3
	1	9.5	-12.9	-3.7	-9.6	-8.9	-12.9	-11.5	8.1	3.2	1.3	-3.0
	2	26.5	16.1	16.3	16.1	24.7	19.5	19.5	24.9	26.3	16.2	15.5
	4	-19.4	9.1	57.7	48.3	38.8	31.0	39.4	39.1	47.0	37.3	37.1
	5	-78.8	-38.1	101.3	81.6	36.1	26.6	52.2	75.3	88.6	57.2	56.7
	av.^2^	30.2	19.6	41.6	36.4	28.3	23.4	30.3	34.4	38.8	26.6	26.5
9	$\bar{\lambda }$	4.4	-9.9	9.5	3.2	11.8	0.8	7.2	19.7	18.2	6.3	2.5
	1	22.4	-22.4	-3.8	-13.7	-6.9	-18.5	-12.7	21.6	8.8	2.9	-4.2
	2	16.6	-17.5	-6.8	-10.0	0.5	-11.4	-6.2	16.1	11.0	-0.7	-3.1
	8	-85.6	-16.4	139.4	111.7	80.8	77.8	104.4	87.5	110.1	86.5	82.2
	9	-97.9	-35.0	270.1	217.5	133.2	134.0	190.5	184.1	217.0	133.4	131.7
	av.	39.9	16.6	68.4	57.3	48.8	45.1	56.9	54.0	61.9	40.0	39.1
**Heritabilities**												
5	1	-1.0	-9.1	-4.4	-7.8	-6.9	-8.3	-7.9	-0.7	-2.9	-1.9	-3.7
	2	0.6	-3.4	0.0	-1.7	-1.1	-2.0	-1.7	2.2	1.4	-0.2	-1.4
	4	0.9	0.2	3.2	2.2	2.5	2.0	2.1	4.1	4.3	1.0	0.5
	5	1.1	1.2	4.6	3.6	3.7	3.2	3.4	5.1	5.4	1.6	1.3
	av.	0.8	3.1	2.7	3.1	3.0	3.1	3.1	3.0	3.4	1.0	1.4
9	1	-0.5	-15.5	-6.8	-11.3	-7.6	-12.3	-10.0	0.9	-3.6	-2.0	-5.4
	2	-0.1	-11.7	-4.8	-7.8	-4.5	-8.5	-6.6	1.9	-1.3	-1.8	-4.9
	8	2.2	2.5	7.5	6.0	7.5	5.7	6.8	8.2	8.6	2.9	2.2
	9	2.5	3.5	8.6	7.2	8.4	6.9	7.9	9.1	9.6	3.5	2.9
	av.	1.3	5.4	4.8	4.8	4.9	5.0	4.9	5.4	5.1	1.9	2.5

Data for *s *= 100 sire families; using different penalties (see text for notation) and population values to determine the tuning factor (strategy V∞)

^1^Number of traits

^2^Average of all *q *absolute values

Penalties on matrix divergence clearly acted in a different manner to penalties on canonical eigenvalues, although PRIAL in **Σ***_G _*were comparable. For $P_{\Sigma }$, the upwards bias in *λ*_1 _was of a similar magnitude and individual estimates exhibited the same pattern (Figure 3) as for unpenalized REML estimates, while penalization predominantly affected the smallest values. This was due to $P_{\Sigma }$ being approximately proportional to the reciprocal of *λ_i_*. Shrinking genetic correlations towards their phenotypic counterparts $\left(P_{\rho }\right)$ yielded the least relative bias in estimates of the leading canonical eigenvalues.

However, it should be stressed that bias in estimates of eigenvalues does not directly translate into bias in the corresponding covariance components or genetic parameters. Eigenvalues of sample covariance matrices are systematically over-dispersed and biased, but the sample covariance matrix is an unbiased estimator e.g. [3]. REML estimates are biased, however, because estimates are constrained to the parameter space. This implies that for scenarios for which no constraints are needed, no bias is notable. Table 3 gives the mean bias in estimates of selected heritabilities (*h*^2^). Without penalty, a slight bias in estimates that corresponded to the highest and lowest population values was evident, arising from constrained estimation. Penalized estimation biased estimates of *h*^2^, with the pattern of biases and differences between penalties similar to those observed for *λ_i_*. For instance, for $P_{\Sigma }$ the smallest *h*^2 ^were substantially biased upwards, while estimates for the largest values were similar to those from unpenalized analyses. Penalties on the canonical eigenvalues resulted in marked underestimates of the highest *h*^2^. Taking the average of absolute deviations across traits yielded the lowest values for $P_{\rho }$ and $P_{\rho }^{2}$, only slightly higher than for unpenalized estimates, whilst mean absolute differences for the other penalties were about twice as high (Table 3).

**Figure 4:**
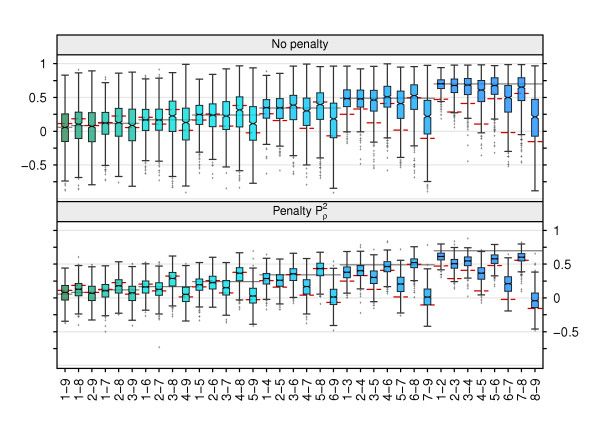
**Distribution of estimates of genetic correlations between traits *i *and *j *(*i*-*j*) across replicates for case T-*VI***. Data for *s *= 100 sires and using strategy V∞ to determine tuning factors; horizontal bars show population values for genetic (solid lines) and phenotypic (dashed lines) correlations.

The effects of penalized estimation on estimates of genetic correlations are illustrated in Figure 4 for case T-*VI *(with population *h*^2 ^of 2 × 0.5, 0.2, 2 × 0.15, 2 × 0.1 and 2 × 0.05) and *s *= 100. Shown is a box-and-whisker plot of individual estimates across replicates, with correlations in ascending order of their population values, depicted by horizontal bars. Not surprisingly for such small samples, unpenalized estimates were subject to substantial sampling variation and were most variable for pairs of traits with lowest *h*^2^. Again, unpenalized estimates were clearly biased due to constraints on the parameter space, with mean deviations from the population values ranging from -0.504 (8-9) to 0.035 (3-8) and a mean, absolute bias across replicates of 0.064. Penalization dramatically reduced the spread of estimates, but increased bias to a range of -0.734 (8-9) to 0.103 (4-8), with a mean absolute value of 0.142. In all cases, genetic correlations were shrunk towards the corresponding phenotypic correlations (population values shown as dashed horizontal lines). In spite of the increase in bias, penalized estimation reduced the loss in the estimate of **R***_G _*by 77.3%. The corresponding value for **Σ***_G _*was less, 58.1% for V∞, i.e. this was a scenario for which penalization was less effective (Figure 1). Across all cases, the mean absolute bias in estimates of genetic correlations for unpenalized estimates for *s *= 100 was 0.046 for *q *= 9 and 0.033 for *q *= 5. Penalized estimation increased this value by a factor of 2 to 3. Again, there was a tendency for the bias to be most pronounced for penalties that were imposed directly on the canonical eigenvalues.

## Discussion

An extension of current, standard methodology to estimate genetic parameters in a mixed model framework has been outlined that has the scope to yield 'better' estimates, especially for multivariate analyses that comprised more than just a few traits. This is achieved by penalizing the likelihood, the penalty being a function of the parameters that is aimed at reducing sampling variation. A number of suitable penalties were investigated, with emphasis on those that 'borrow strength' from estimates of phenotypic covariance components, which are typically estimated much more accurately than their genetic counterparts. All penalties presented have a Bayesian motivation, i.e. they can be derived assuming certain prior distributions for covariance matrices or their eigenvalues.

Simulation results demonstrate that substantial loss reductions, i.e. the (average) difference between true and estimated covariance matrices, can be achieved through penalized estimation. As expected, this reduction in loss is at the cost of increasing bias, over and above that introduced by constraining estimates to the parameter space in standard REML analyses. The magnitude and direction of the additional bias depend on the population parameters and penalty applied but in general, penalization leads to reduced estimates of the highest heritabilities and increases estimates of the smallest heritabilities while estimates of genetic correlations are reduced in absolute value. With comparable (or better) reductions in loss to other penalties, those which shrink the genetic towards the phenotypic correlation matrix ($P_{\rho }$ and $P_{\rho }^{2}$) appeared to result in least bias.

Penalized REML estimation for penalties on canonical eigenvalues is best implemented by parameterising to the elements of the canonical decomposition, **Λ **and **T **[15]. In contrast to implementations for standard REML algorithms (which usually parameterize to the elements of the Cholesky factors of the covariance matrices to be estimated), this yields non-zero derivatives of all covariance matrices with respect to all parameters. Furthermore, initial experience with this parameterization has shown that it resulted in slower convergence rates than estimation of covariance matrices or their Cholesky factors, similar to results by [30]. Moreover, extension to models with additional random effects and penalties on their covariance matrices is not straightforward. However, estimation with penalties on matrix divergence is readily carried out using standard parameterizations, for which calculation of derivatives of the penalty is the only modification to existing REML algorithms required. Furthermore, with this approach penalties on additional covariance matrices can easily be imposed, provided appropriate tuning factors are available.

CV is a widely used technique to estimate the tuning factor in regularization problems from the data at hand. For our application, however, it was found to be only moderately successful, with errors in estimating *ψ *limiting achieved PRIAL and increasing the proportion of replicates for which penalization was detrimental. These errors appeared especially important for larger samples, i.e. in small samples any degree of penalization is likely to have a substantial effect, while over-penalization becomes more harmful as sample size increases. An added problem with CV for data with a genetic family structure is that of representative sampling of data subsets. In our setting, assigning whole sire families to individual folds was a natural choice and yielded higher PRIAL values than splitting families evenly across folds. In practical data sets with arbitrary relationships and fixed effects, choices are less obvious and guidelines to good sampling strategies in a mixed model setting are scarce.

Moreover, CV is laborious and increases the number of analyses required by orders of magnitude. A sequential search for the optimal tuning factor was used in our simulation study. A more efficient strategy would be to use one of the many structured optimization methods available, e.g. a quadratic approximation of the average likelihood from the validation sets. However, this relies on the 'validation' curves to be smooth, increasing monotonically to a maximum and then decreasing again. This was not always the case in the simulations presented - some jagged curves were encountered, in particular for the smallest sample sizes. Presumably this was due to likelihood surfaces being very flat around their maxima, resulting in inaccurate location of these points. Use of such techniques was thus disregarded here.

Fortunately, choice of *ψ *based on the decrease in the unpenalized likelihood from its maximum at *ψ *= 0 can result in penalized estimates that are closely related to those which would be obtained if population values were known. As demonstrated, such strategies yielded average loss reductions for estimates of the genetic covariance matrix that were substantially higher than loss reductions obtained when estimating *ψ *by CV, and loss reductions comparable to those achieved when using knowledge of the population parameters for some penalties. Choosing the limit to the change in likelihood so that it was just not statistically significant appeared to be a sensible choice to select a mild degree of penalization. Although this choice did not perform quite as well for individual cases where all population canonical eigenvalues were very similar, this is a scenario which is unlikely to be of practical relevance in quantitative genetic applications.

Work so far has considered a balanced scenario, in which all traits in a multivariate analysis were measured for all individuals. However, we often have a substantial discrepancy between the number of observations available for different traits. For instance, we may have a number of traits recorded on a substantial number of individuals whilst records for difficult to measure traits are available for a small subset only. It is then necessary to penalize parts of the genetic covariance matrix corresponding to such groups of traits differently. An extension of the penalties on the divergence between genetic and phenotypic matrices allowing this can be derived assuming a generalized inverse Wishart prior distribution, and will be considered in future work.

Even with today's computational resources, there are problems for which analyses that consider all traits of interest are not feasible, so that elements of the complete covariance matrix have to be obtained through a series of analyses of subsets of traits. This yields multiple estimates of variance and some covariance components, which need to be pooled whilst ensuring the resulting matrix is positive definite. Typically, this is done by considering one matrix at a time, using methods such as 'iterative summation of expanded part matrices' [31], or by combining simple averaging of components with a regression of the eigenvalues of the resulting matrix towards their mean so that they are positive. Results from this study suggest that considering all matrices of interest simultaneously when pooling estimates from analyses of subsets, together with some shrinkage towards their sum, may be advantageous.

## Conclusions

Penalized maximum likelihood estimation provides the means to 'make the most' of limited and precious data and facilitates more stable estimation for multi-dimensional analyses, even when samples sizes are larger. We anticipate that penalized maximum likelihood estimation will become part of our everyday toolkit, as truly multivariate estimation for quantitative genetic problems becomes routine. At the present state of knowledge, a mild penalty on the divergence of the genetic from the phenotypic correlation matrix, chosen on the basis of the change in likelihood from an unpenalized analysis, appears the most suitable option for practical applications.

## Competing interests

The author declares that she has no competing interests.

## Authors' contributions

KM carried out all the tasks associated with this paper. All authors read and approved the final manuscript.
